# Submucosal dissection and debridement for migratory esophageal foreign body removal in an elderly patient with acute presentation: A case report

**DOI:** 10.1097/MD.0000000000046915

**Published:** 2026-01-23

**Authors:** Lei Zhang, Nana An, Xiuli Zheng, Limian Er

**Affiliations:** a Department of Endoscopy, The Fourth Hospital of Hebei Medical University, Shijiazhuang, Hebei, China.

**Keywords:** endoscopic submucosal dissection, endoscopic ultrasound, esophageal foreign body, multidisciplinary team

## Abstract

**Rationale:**

Migratory esophageal foreign bodies (EFB) with submucosal migration and infection pose significant challenges in high-risk elderly patients. While standard endoscopy manages superficial cases, guidelines lack strategies for deeply embedded EFBs with infection risks. This study aims to evaluate endoscopic submucosal dissection (ESD) combined with en bloc extraction and precision debridement as a nonsurgical approach for octogenarians, specifically addressing infection control issues in endoscopic EFB management.

**Patient concerns:**

In this report, we present a case of an 84-year-old woman who developed acute dysphagia accompanied by high fever following fish bone ingestion. Initial endoscopic examination revealed a distal esophageal erosion (15 mm), with no direct observation of a foreign body.

**Diagnoses:**

A computed tomography scan revealed proximal esophageal hyperdensity (non-perforating), later confirmed by endoscopic ultrasound as a 5.4 mm hypoechoic lesion with a hyperechoic core, indicating submucosal migration of the fish bone. Accompanying pyrexia (38.7 °C) and leukocytosis (white blood cells 12.0 × 10⁹/L) suggested inflammatory progression.

**Interventions:**

A structured multidisciplinary team approach was implemented, culminating in single-session ESD with en bloc foreign body extraction and precision debridement under monitored anesthesia care. The patient received intravenous cefoperazone sodium and sulbactam sodium for 48 hours post-procedure.

**Outcomes:**

The en bloc EFB extraction via ESD (35 minutes) achieved complete debridement without bleeding or perforation. Postoperative antibiotics (48-hour course) resolved fever (38.7 °C → 36.8 °C) within 24 hours and normalized leukocytosis (white blood cells 12.0 → 6.8 × 10⁹/L). Oral intake progressed from liquids (day 3) to soft solids (day 7), with telemedicine follow-up confirming sustained dysphagia resolution. Discharge occurred on day 5 with no hematemesis/melena, and 30-day monitoring revealed no complications (strictures, aspiration), demonstrating procedural safety for high-risk octogenarians.

**Lessons:**

This preliminary experience highlights the potential utility of ESD for foreign body clearance alongside selective debridement in managing migratory EFB complicated by localized infection and acute dysphagic manifestations in octogenarian patients, thereby proposing a feasible nonsurgical alternative approach.

## 1. Introduction

Esophageal foreign bodies (EFB) impacted in the esophagus should be removed within 24 hours because delay decreases the likelihood of successful removal and increases the risk of complications.^[1]^ The removal of migratory EFB poses challenges due to their complexity and concealment. The selection between surgical intervention and endoscopic resection requires comprehensive clinical evaluation, with risks of severe complications such as perforation, cervical abscess, mediastinal abscess, dysphagia, and fever.^[2,3]^

We present a rare case of migratory EFB in an octogenarian, complicated by acute dysphagia and high-grade fever. Successful endoscopic submucosal dissection (ESD) with targeted debridement achieved complete foreign body retrieval and resolution of acute symptoms.

## 2. Case presentation

An 84-year-old female presented with acute dysphagia post fish bone ingestion on April 25, 2025. There was no documented history of hypertension, coronary artery disease, diabetes, dysphagia, or gastrointestinal symptoms (including nausea/vomiting) in the medical records. The patient reported no familial history of esophageal carcinoma in first-degree relatives. Odynophagia restricted to cervical and superior mediastinal areas was documented, with negative findings for tenderness on palpation, pain radiation, or cervicothoracic subcutaneous air on clinical evaluation.

**Figure 1.**
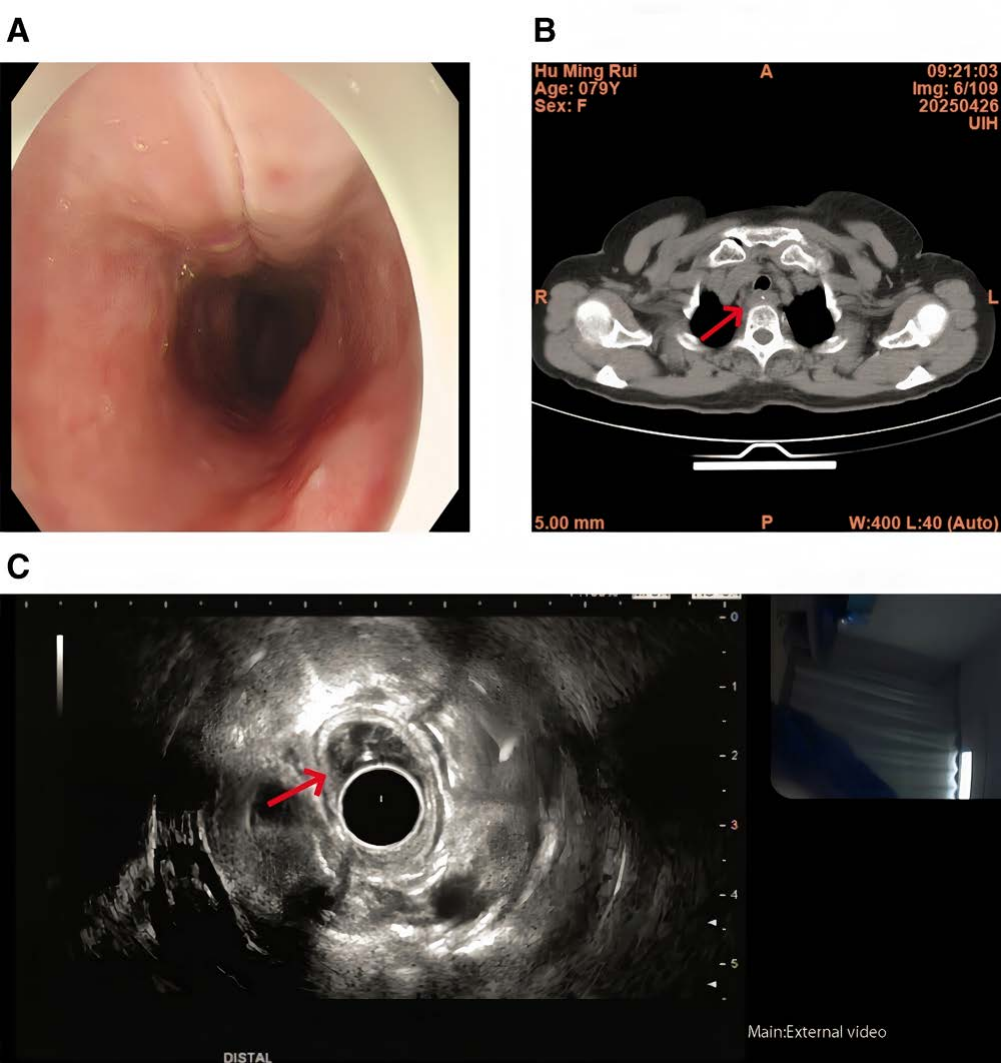
. (A) Esophagogastroduodenoscopy finding. (B) Cervical/thoracic CT revealed a hyperdense shadow in the proximal esophagus, with no radiographic signs of perforation. (C) Endoscopic ultrasound (EUS) identified a 5.4 mm thick heterogeneous hypoechoic area containing a hyperechoic core. CT = computed tomography.

Standard gastroscopy revealed mucosal edema accompanied by a 15 mm longitudinal erosion at 20 cm from the incisors, though no definitive foreign body was visualized endoscopically (Fig. 1A). Cervical/thoracic computed tomography (CT) performed on April 26 revealed a hyperdense shadow in the proximal esophagus, with no radiographic signs of perforation (Fig. 1B). Subsequent endoscopic ultrasound conducted on April 27 identified a 5.4 mm thick heterogeneous hypoechoic area containing a hyperechoic core (Fig. 1C), confirming submucosal impaction with perilesional inflammation. The patient manifested pyrexia with a recorded body temperature of 38.7 °C (101.7 °F), accompanied by leukocytosis (white blood cells [WBC] 12.0 × 10^9^/L, neutrophil predominance 85%).

The constellation of CT and endoscopic findings supports the diagnosis of transmural fish bone migration. The possibility of complete transesophageal passage into the gastric lumen cannot be excluded. Definitive management should be guided by multidisciplinary consultation, with therapeutic options that may include either surgical exploration or minimally invasive endoscopic management combining submucosal dissection with targeted debridement.

Given the patient’s advanced age and elevated surgical risks associated with exploration, a structured multidisciplinary team approach was ultimately implemented. This culminated in single-session ESD under monitored anesthesia care, achieving en bloc foreign body extraction coupled with precision debridement.

**Figure 2.**
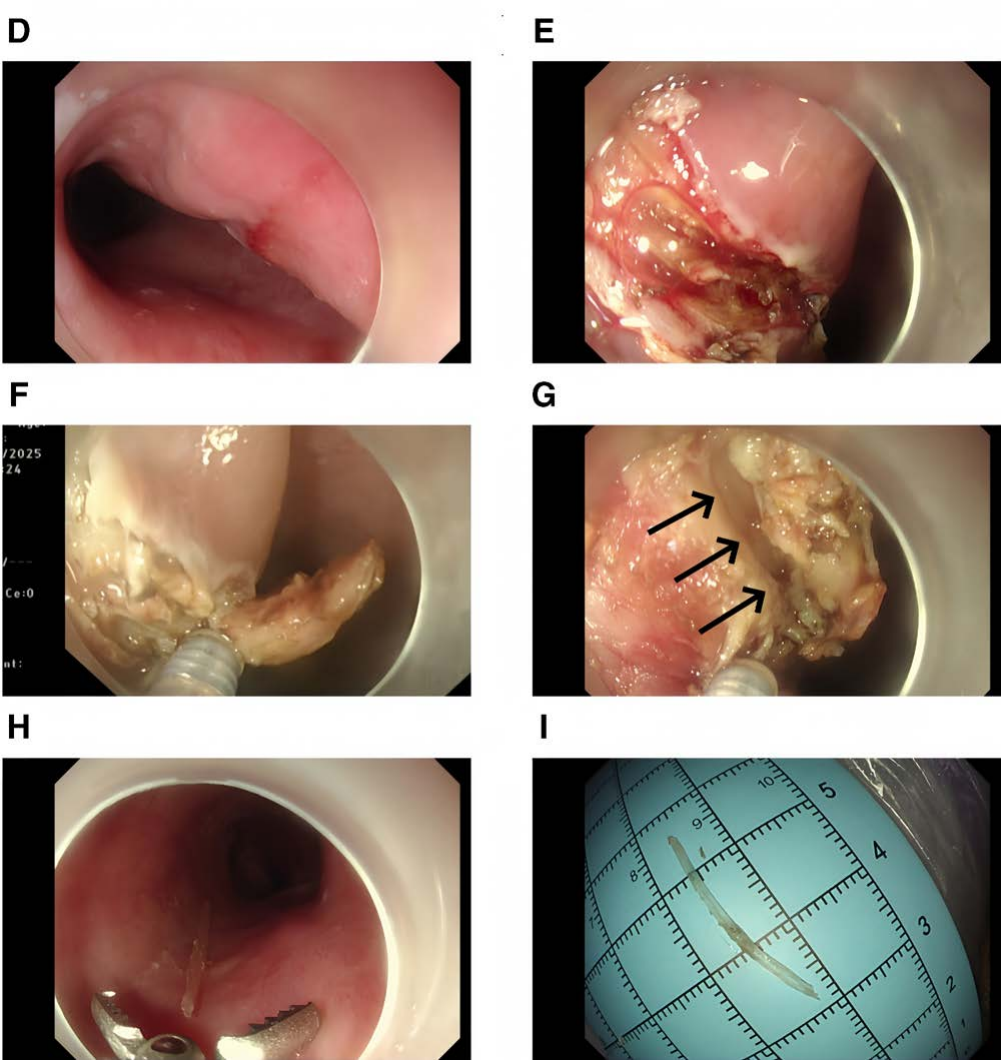
(D and E) Incision along the longitudinal axis of the esophagus. (F) Systematic debridement of necrotic tissues. (G) Exposed fish bone in the submucosal layer (arrow indicates location). (H and I) Length of fishbone measured after removal, which was approximately 25 mm in length.

Procedural steps included:

1. Mucosal incision using mucosal incision knife (Micro-Tech, Nanjing) at the proximal marker without injection (Fig. 2D and E).
2. Debridement of necrotic tissues (Fig. 2F).
3. Identification of vertically embedded foreign body (25 mm × 1.5 mm fish bone) in submucosal layer (Fig. 2G).
4. Complete extraction using foreign body forceps (Fig. 2H and I).
5. Repeat debridement of residual necrotic tissue, nasogastric tube monitoring.
6. Postoperative patient management: the en bloc extraction was completed within 35 minutes of procedural time, achieving complete submucosal debridement without intraoperative complications (bleeding/perforation). Postoperative prophylactic antibiotics (cefoperazone sodium and sulbactam sodium) administered for 48 hours effectively controlled localized infection, with no progression of infectious signs. Fever completely resolved within 24 hours (38.7 °C to 36.8 °C), and leukocytosis normalized by discharge (WBC 12.0 → 6.8 × 10⁹/L). No hematemesis or melena occurred during hospitalization. Functional recovery milestones included tolerance of liquid intake by postoperative day 3, transition to a semiliquid diet preceding discharge on day 5, and advancement to soft solids by day 7, with telemedicine follow-up confirming sustained resolution of dysphagia. No readmissions or complications (e.g., strictures, aspiration) were observed during the 30-day follow-up period, underscoring the procedural safety profile in this high-risk cohort.

The study was carried out after the protocol was approved by the Ethics Committee of the Fourth Hospital of Hebei Medical University. The patient gave written informed consent for the publication of clinical details and images.

## 3. Discussion and conclusions

Foreign body impaction predominantly occurs at esophageal sites,^[4]^ among which fish bones constitute the most prevalent impacted objects.^[5]^ Extensive clinical evidence has well-documented the progressive complications secondary to fish bone ingestion, including esophageal perforation, pyrexia, acute dysphagia, mediastinitis, mediastinitis-associated thoracic aortic pseudoaneurysm, and rare migration into pulmonary structures, collectively contributing to elevated mortality rates in untreated cases.^[3,6–9]^ While endoscopic retrieval remains the first-line therapeutic intervention with 96.45% success rate,^[5]^ its efficacy diminishes significantly in radiographically confirmed migratory foreign bodies. They systematic review identified 23 documented cases of dynamic esophageal migration, with 21 (91.3%) ultimately requiring transcervical surgical approaches, predominantly via the cervical esophageal-triangle pathway.^[3]^

This elderly female patient presented with acute dysphagia accompanied by high fever within 48 hours. Through multidisciplinary collaboration utilizing neck/chest CT and endoscopic ultrasound, combined with anesthesia support, complete removal of the fish bone was achieved via submucosal dissection and debridement of necrotic tissue under general anesthesia. This case involves a migratory EFB with confirmed submucosal migration of a fish bone accompanied by localized inflammatory response. While endoscopic retrieval of migratory esophageal foreign bodies has been documented in clinical reports, existing literature primarily describes cases involving middle-aged male patients without acute dysphagia or febrile presentation.^[10]^

The patient’s febrile presentation and acute dysphagia are believed to result from dual pathological mechanisms: foreign body migration through the lamina propria inducing focal inflammatory cascade (WBC 12.0 × 10^9^/L), and subsequent submucosal inflammatory thickening (5.4 mm vs normal 1.2 mm) confirmed by endoscopic ultrasonography.

This study also has several limitations that must be acknowledged. Single-case limitation: observations derive from an isolated octogenarian case, necessitating validation through larger studies to confirm generalizability. Resource dependency: the ESD success relied on specialized endoscopic expertise and multidisciplinary coordination, which may limit applicability in nonspecialized settings. Short-term follow-up: absence of long-term data precludes evaluation of delayed complications (e.g., strictures).

In conclusion, this preliminary experience highlights the potential utility of endoscopic submucosal dissection for foreign body clearance alongside selective debridement in managing migratory EFB complicated by localized infection and acute dysphagic manifestations in octogenarian patients, thereby proposing a feasible non-surgical alternative approach.

## Acknowledgments

The authors thank the patient for providing informed consent for publication of this case report and associated images.

## Author contributions

**Data curation:** Lei Zhang, Nana An, Limian Er.

**Formal analysis:** Lei Zhang, Nana An, Xiuli Zheng, Limian Er.

**Writing – original draft:** Lei Zhang, Limian Er.

**Writing – review & editing:** Limian Er.
